# Medical Science and Ethics in War

**Published:** 1916-10-14

**Authors:** 


The Hospital

The Workers' Newspaper of Administrative Medicine and Institutional Life, Administration, National Insurance and Health.

No. 1584, Vol. LXI. SATURDAY, OCTOBER 14, 1916. PRICE ONE PENNY.

### MEDICAL SCIENCE AND ETHICS IN WAR.

Whoever else may be right or wrong when engaged in active military proceedings, the medical man at least would seem to have an unembarrassed course. A comrade of those who fight, he is yet not of the battle; and though he may suffer blows, he never inflicts them. His plain duty is to give help and succour to those who need them, and his mission is one not of destruction but of mercy. Hence, while many anxious and delicate ethical questions may arise for combatants, none such would appear to apply to the medical officer. Whatever indictment may be framed against war in general, or against some individual war in particular, his conscience surely escapes agitation. Recent experience has indeed shown that some few persons afflicted with a peculiar type of mind object on what are called conscientious grounds to render even medical aid to the wounded. But these are so rare and exceptional that they may safely be disregarded. The broad fact remains that the medical officer on the battlefield, whatever be the merits of the contest, stands secure in the respect and regard of human sympathy and support. And not the least engaging feature of his work is his readiness as a matter of duty and goodwill to render aid alike to friends and foes.

But while for the individual occupied with the care of sick and wounded soldiers there is a mental position free from questionings, it is not possible to say that the whole of the medical position relative to war is unclouded by hesitations and doubts. In particular, a discussion may arise whether war does or does not alter the obligation of honour—accepted naturally in time of peace—to disclose for the benefit of all nations methods of treatment which in the circumstances of the hour have a special and helpful application. The natural inclination of the medical man, founded upon a well-established tradition, is certainly to make no secret of what may help to lessen suffering and to save life. He is not accustomed to recognise national enmities or rivalries as influences which justify neglect of the larger claims of humanity, and therefore his disposition must be to continue in war what he has been accustomed to practise in peace. The difficulty arises when it is recognised that the new or improved methods of treatment which are here suggested as possibilities may, if publicly proclaimed, be helpful to the enemy. Moreover, and this is the crux of the matter, the help may be of such a nature that wounded men who would otherwise remain incapacitated, perhaps for the remainder of the war, may be enabled more or less promptly to rejoin the fighting ranks. In a word, the step here contemplated may strengthen the enemy as a combatant force by increasing the number of his active soldiers. Hence, whatever admiration it may merit as a proceeding in accordance with humanitarian motives and professional traditions, it is capable of being represented as substantially antipatriotic and opposed to the national interest. Still more, such an action, it may be argued, is not really humanitarian, for by helping to increase the enemy's resisting power it may prolong the war and may thus in the end cause more suffering than it relieves. Here plainly, then, is a case of conscience, and the issue is no mere academic debate. On the contrary, it is actual and existent. Unless rumour is indeed the lying jade which the moralists proclaim her to be, disciplined efforts have been put forward in Germany to prevent the leakage of medical and surgical information which might possibly be of service in the treatment of wounded opponents. And it would be strange if Germany were the only belligerent nation in which the question has arisen. We will not say that it has arisen in this country, but obviously it may arise, and there is thus need to be provided in advance with an answer.

Now, were the situation to which a rule had to be applied an ideal one, there would be no difficulty either in substance or in terms. If all would adopt the practice, manifestly the humane course would be for free publication of medical improvements and discoveries in war even as in peace. Injured and stricken men of all nationalities would reap the advantage. It is more than possible that the number of sick and wounded who would be able to return to combatant service would be increased. But many others for whom no such return is possible would enjoy more effective treatment, and would thus be saved suffering and perhaps permanent disability; and the chance of increase in combatant force would be equal on the two sides. To this end, it seems to us, the influence of the medical profession ought to be directed, so that the practice here sketched could be included among the laws of war. Anything less than such a practice cannot fail to be repugnant to the public opinion of the profession which regards with contempt the concealment for selfish ends of knowledge which may lessen human suffering. But for the moment we have to deal with a situation which is anything but ideal. The free nations of Europe are fighting for their very existence. Their principal enemy has shown little scruple in the employment of means to secure his ambitions. Abundant circumstances justify the suspicion that he will keep as a strict secret anything which would facilitate the re-employment of any of our wounded men as combatants. Must we not in justice to our own soldiers adopt a similar practice? It is not without reluctance that we feel compelled to answer this question in the affirmative. We would not for a moment allow that whatever the enemy does we are justified in doing, and we feel quite sure that the national conscience is in harmony with this view. But the question now at issue is not of reprisals in the strict sense of the term. No one would allow that wounded men should be killed in order to prevent their possible return to the fighting ranks. The suggestion rather is whether we should tell the enemy of means which would facilitate such return. It is easy and tempting to be generous and chivalrous, but we cannot cultivate these virtues at the expense of our soldiers. Our first obligation is to our fighting Services, and there is no ethical bond upon us to multiply their opponents and thus to increase their peril.

